# The burden of Lipoprotein(a) [Lp(a)] in Africa: A systematic review

**DOI:** 10.1371/journal.pone.0356490

**Published:** 2026-08-26

**Authors:** Lambert Tetteh Appiah, Florence Koryo Akumiah, Jacob Solomon Idan, Gordon Manu Amponsah, Solomon Gyabaah, Birgit Agyeiwaah Baah, Frederick Kwarteng Owusu, Benedict Kusi Ampofo, Robert Winfred Eshun, Oladotun Victor Olalusi, Bert-Jan van den Born, Charles Agyemang

**Affiliations:** 1 Division of Cardiology of the Department of Medicine Kwame Nkrumah University of Science and Technology, Kumasi, Ghana; 2 Department of Medicine, Komfo Anokye Teaching Hospital, Kumasi, Ghana; 3 Department of Public & Occupational Health, University of Amsterdam, Amsterdam, UMC, Amsterdam, The Netherlands; 4 National Cardiothoracic Centre, Accra, Ghana; 5 Department of Medicine, Korle Bu Teaching Hospital, Accra, Ghana; 6 Department of Epidemiology and Biostatistics, School of Public Health, Kwame Nkrumah University of Science and Technology, Kumasi, Ghana; 7 Department of Physiology, School of Medical Sciences, Kwame Nkrumah University of Science and Technology, Kumasi, Ghana; 8 Public Health Unit, Komfo Anokye Teaching Hospital, Kumasi, Ghana; 9 Department of Neurology, University College Hospital Ibadan Nigeria, Ibadan, Nigeria; 10 Hussman Institute for Human Genomics, University of Miami Miller School of Medicine, Miami, Florida, United States of America; 11 Department of Vascular Medicine, Amsterdam Cardiovascular Sciences, Amsterdam UMC, University of Amsterdam, Amsterdam, The Netherlands; 12 Department of Public & Occupational Health, University of Amsterdam, Amsterdam, UMC, Amsterdam, The Netherlands; University of Ghana Medical School, GHANA

## Abstract

**Background:**

Lipoprotein(a) [Lp(a)] is a low-density lipoprotein-like particle covalently bound to apolipoprotein(a) and encoded by the *LPA* gene. Elevated plasma levels of Lp(a) are a well-established, independent, and causal risk factor for cardiovascular disease. While the epidemiology of Lp(a) has been extensively mapped in non- African populations, the burden and distribution of Lp(a) in African populations remain under-characterized despite its unique genetic, phenotypic, ethnolinguistic and ancestral diversity. The primary objective was to synthesize existing literature to determine the prevalence and distribution of elevated Lp(a) levels in apparently healthy African individuals residing on the African continent.

**Methods:**

This systematic review included observational studies (cross-sectional, cohort, and case-control) involving apparently healthy adults (≥18 years) in Africa. The review systematically searched PubMed/MEDLINE, Embase, Scopus, African Journals Online, and Google Scholar without language or date restrictions to capture the full breadth of historical and contemporary data. Data were extracted using a standardized form capturing study characteristics, population demographics, assay methods and quantitative outcomes. Methodological quality was assessed using the Newcastle-Ottawa Scale.

**Results:**

A total of 17 studies met the inclusion criteria, representing populations from West Africa (Nigeria, Ghana) Central Africa (Cameroon), Southern Africa (South Africa, Zimbabwe), and North Africa (Morocco, Tunisia). Studies in Ghana reported prevalence rates of elevated Lp(a) (>30 mg/dL) between 30–61%. Lp(a) mean levels ranged from 12.7 mg/dL to 54.5 mg/dL depending on the cohort and assay used. Females exhibited higher Lp(a) levels than males across most African cohorts. Clinically, elevated Lp(a) was associated with hypertension, type 2 diabetes mellitus, ischemic stroke, myocardial infarction and anti-retroviral drug use.

**Conclusion:**

Elevated Lp(a) is a common, genetically determined trait in Africa rather than a rare dyslipidemia. Far from being a benign variant, it acts as a significant amplifier of cardiovascular risk, particularly in the presence of rising incident rates of hypertension and diabetes. Addressing this burden requires the adoption of standardized, isoform-independent assays and the development of population-specific risk thresholds to guide targeted preventive cardiovascular care.

## Introduction

Cardiovascular diseases (CVD) remain the preeminent cause of morbidity and mortality worldwide [1]. The burden is disproportionately borne by low- and middle-income countries (LMICs) [2,3] which is attributable to population expansion and a myriad (or complex interplay) of genetic, environmental and lifestyle factors [4]. Lipoprotein(a) [Lp(a)] has emerged over the last decade as a genetically determined and one of the prevalent risk factors of CVD [1,5]. Structurally, Lp(a) is a unique lipoprotein subclass that resembles a low-density lipoprotein (LDL) particle in its lipid composition and the presence of apolipoprotein B-100 (apoB-100) [1]. However, it is distinguished by the presence of a second apolipoprotein(a) [apo(a)], which is covalently linked to the apoB-100 moiety via a single disulfide bond [5]. It shares a high degree of structural homology with plasminogen [1]. This unique assembly confers upon Lp(a) a pro-atherogenic properties of LDL due to its cholesterol content whiles the apo(a) moiety adds distinct pro-thrombotic and pro-inflammatory predilection [1].

Plasma Lp(a) concentrations are highly heritable and genetically determined [5]. Genetic factors account for approximately 70% of the inter-individual variation observed in global populations [1]. The apo(a) protein is encoded by the *LPA* gene located on the long arm of chromosome 6 (6q26-27) [6]. High copy number variation in the Kringle IV type 2 domain is the molecular basis for the extreme size heterogeneity of the apo(a) protein (isoforms) observed in the human population [1,6]. The KIV-2 copy number accounts for a substantial proportion of the variance in Lp(a) levels in European populations, often exceeding 70% [1,5,6]. In contrast, it explains a considerably smaller proportion of the phenotypic variance in the African populations [1,6]. This suggests that the genetic architecture underlying Lp(a) regulation is distinct in individuals of African descent. Notable, individuals of African descent have significantly higher median Lp(a) levels compared to Caucasians, East Asians, or Hispanics [6]. Unlike the African continent, where Lp(a) are high but less explained by genetics [1,6], the risk profile of Lp(a) in the Western populations is well-defined. This may pose as a reservoir of latent cardiovascular risk [3]. Existing reviews frequently aggregate data from the western world [6,7] however the burden in Africans remains under-explored and often conflated with data from the diaspora [8].

This systematic review addresses a critical knowledge gap by synthesizing existing data on the prevalence, distribution, and determinants of lipoprotein(a) [Lp(a)] in continental Africa. By characterising the burden, this study hopes to shape understanding, and strengthen the need for region-specific screening and risk stratification guidelines for preventative cardiovascular care.

## Methodology

### Protocol and protocol registration

This systematic review was conducted in strict adherence to the 2020 guidelines and the Cochrane Handbook for Systematic Reviews of Interventions [9]. The review protocol was prospectively registered in the International Prospective Register of Systematic Reviews (PROSPERO) with registration number CRD420250652484 prior to data extraction.

### Information sources and search strategy

A systematic search strategy (see S1 Table) was implemented to identify all relevant literature with the help of an experienced medical librarian. The systematic search was conducted across five major electronic databases. These databases were PubMed/MEDLINE, Scopus, Embase, African Journals Online and Google Scholar. A combination of Medical Subject Headings (MeSH) and free-text keywords was used, with Boolean operators (AND, OR) applied to combine terms related to lipoprotein(a), the African population, and relevant outcomes. Search terms were structured around three core concepts. These were lipoprotein(a) (including “Lipoprotein(a),” “Lp(a),” “apolipoprotein(a),” “LPA,” and “lipoprotein antigen”), population (including “Africa,” “sub-Saharan Africa,” “West Africa,”, “Central Africa,” “East Africa,” “Southern Africa,” “North Africa,” “African,” “Black,” “Adult*,” and “mature individual*”), and outcome or context (including “prevalence,” “healthy,” “control,” “general population,” “epidemiology,” “burden,” and “distribution”). In addition, reference lists of all included studies and relevant review articles were manually screened to identify additional eligible studies, including older or non-indexed publications.

### Eligibility criteria

#### Inclusion criteria.

Studies were eligible for inclusion if they involved adult participants aged 18 years and older residing in any country on the African continent and were classified as an apparently healthy stable outpatient. Apparently healthy stable outpatient adult, in this context, is defined as individuals from the general population who are healthy control groups, or individuals with stable, non-acute chronic conditions and without evidence of acute illness at the time of assessment. These individuals with stable, non-acute chronic conditions included individuals with virologically suppressed HIV, controlled hypertension, controlled diabetes mellitus, or stable post-event cardiovascular patients. The outcome of interest was the quantitative measurement of serum or plasma lipoprotein(a) [Lp(a)] concentration. Studies published in any language were considered, provided an English abstract was available to facilitate screening. Data extraction was restricted to publication date before the year 2026.

#### Exclusion criteria.

Studies focusing exclusively on acutely ill patient populations or unstable chronic conditions, as well as case series, case reports and interventional trials were excluded. In addition, studies that reported results solely as qualitative presence or absence without numerical values were excluded. Conference abstracts, editorials, commentaries, letters to the editor, narrative reviews, systematic reviews, and meta-analyses were excluded. Duplicate publications, studies with insufficient data to extract relevant outcomes, and publications without an English abstract available for screening were also excluded.

### Study selection process

The study selection process followed a two-stage screening approach which was consistent with the PRISMA guidelines [10]. The first stage was title and abstract screening. Two independent reviewers (LTA and JSI) screened the titles and abstracts of all identified records using Rayyan, a web-based systematic review management platform [11]. Records that clearly did not meet the eligibility criteria were discarded. The second stage was a full text review of the selected articles done independently after the screening. The full text of all potentially relevant articles was retrieved and assessed against the inclusion/exclusion criteria. Disagreements between the two reviewers regarding eligibility were resolved through discussion and consensus, or by consultation with a third senior reviewer if necessary.

### Data extraction

Data were extracted from the included studies using a pre-piloted, standardized data extraction form (see S2 Table). Extracted information included study identification details (first author, year of publication, and study title); study setting (country, region classified according to the United Nations geoscheme, urban or rural setting, and specific study location); and study design (cross-sectional, case–control, or cohort). Population characteristics captured included the sample size of the apparently healthy group, age distribution (mean, standard deviation, and range where available), sex distribution (percentage female), and reported ethnicity or tribal affiliation such as Ashanti, Bantu, Khoisan, or mixed ancestry. Detailed information on Lp(a) measurement was extracted, including assay type, commercial assay kit and manufacturer, whether the assay was reported to be isoform-independent or calibrated against a reference standard such as the WHO/IFCC SRM 2B, and units of measurement (mg/dL, mg/L, or nmol/L) [12]. Quantitative outcome data extracted included measures of central tendency (mean with standard deviation or median with interquartile range), reported prevalence of elevated Lp(a) using study-specific thresholds (including >30 mg/dL, >50 mg/dL, >300 mg/L, >500 mg/L, or >75/125 nmol/L), and indicators of distribution such as range or skewness where available. Additionally, reported correlates of Lp(a) levels including age, sex, body mass index, blood pressure, lipid profile components (LDL-C, HDL-C, triglycerides), and genetic polymorphisms were extracted when reported.

### Risk of bias assessment

The methodological quality and risk of bias of the included studies were assessed using the Newcastle–Ottawa Scale, adapted for cross-sectional and case–control study designs [13] (see S3 Table). The Newcastle–Ottawa Scale evaluates study quality across three domains. These are selection, comparability, and outcome [13]. The selection domain (maximum of five stars) assessed the representativeness of the study sample in relation to the target population, the presence of a sample size calculation or justification, and the reporting and adequacy of response rates or the handling of non-respondents. The comparability domain (maximum of two stars) evaluated whether studies appropriately controlled for key confounding variables, particularly age and sex, and for additional relevant factors such as body mass index, smoking status, or renal function where applicable. The outcome domain (maximum of three stars) assessed the validity and reliability of lipoprotein(a) measurement methods and the appropriateness of the statistical analyses used. Based on the total NOS score, studies were classified as low risk (7–9 stars), moderate risk (5–6 stars), or poor quality with high risk of bias (0–4 stars)

### Data synthesis and analysis

Data synthesis and analysis were conducted using a narrative approach. Due to the substantial heterogeneity in study designs (cross-sectional, case-control, and cohort), populations characteristics (healthy controls, blood donors, community samples, and hospital-based cohorts), Lp(a) assay methodologies (ELISA, IRMA, nephelometry, and immunoturbidimetry), and reporting units (mg/dL, mg/L, µmol/L, and nmol/L), a formal meta-analysis was deemed inappropriate and potentially misleading. This study acknowledges that WHO/IFCC recommends reporting Lp(a) in nmol/L to eliminate bias from apo(a) isoform size variability [12], however, given that majority of included studies reported exclusively in mass units (mg/dL or mg/L), conversion to nmol/L would have introduced additional uncertainty without improving comparability with the existing African literature base. For harmonization and comparative purposes, lipoprotein(a) concentrations reported in various units (mg/L, µmol/L, or nmol/L) were converted to mass units (mg/dL) using standard conversion factors (mg/L ÷ 10; nmol/L ÷ 2.15; µmol/L converted to nmol/L × 1000, then ÷ 2.15) respectively, and results are presented primarily in mg/dL alongside original values [14]. Although it is recognized that such universal conversion factors may introduce imprecision due to inter-individual variability in apolipoprotein(a) molecular weight, this approach was adopted to facilitate consistency in reporting across studies. Findings were synthesized narratively and grouped by United Nations geoscheme region (West, East, Central, Southern, and North Africa) to explore regional patterns and potential genetic substructures. Study characteristics, converted lipoprotein(a) levels, prevalence estimates, and risk of bias assessments were summarized using structured tabular formats to support cross-study comparison. This approach aligns with best-practice guidance for synthesizing highly heterogeneous observational data [15].

## Results

### Search results and study selection

**Fig 1:**
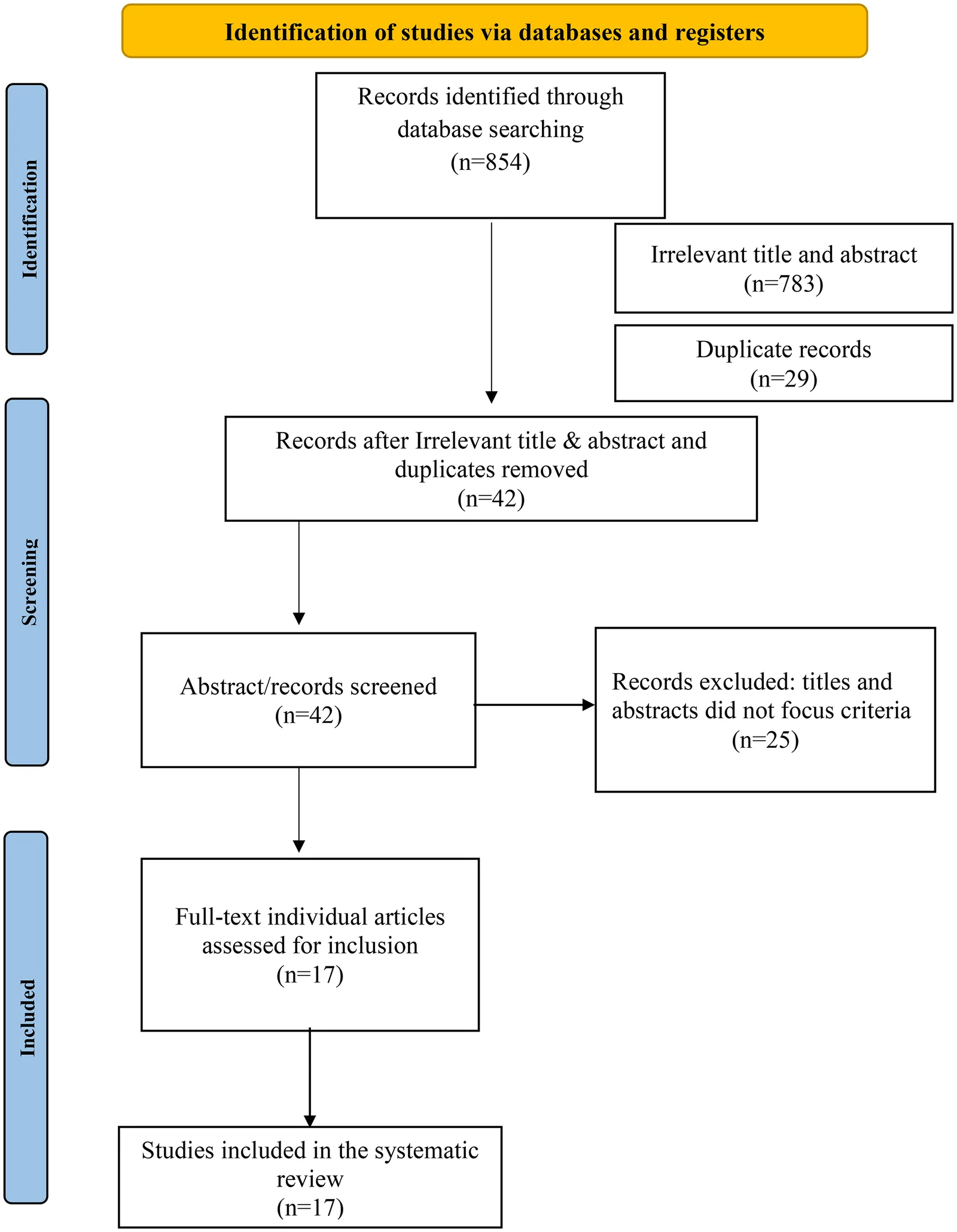
PRISMA flow diagram of study selection. Database searching identified 854 records. After removal of 783 records with irrelevant titles or abstracts and 29 duplicate records, 42 records were screened. Twenty-five records were excluded, leaving 17 full-text articles that met the eligibility criteria and were included in the systematic review. PRISMA, Preferred Reporting Items for Systematic Reviews and Meta-Analyses.

A total of 854 records were retrieved from the initial database search (Fig 1).

After removing duplicates and screening titles and abstracts, 42 articles were selected for full-text review. Of these, 25 studies were excluded because the study population was not resident in Africa (n = 6), data were reported in aggregated form for Black populations without distinction between continental African and diaspora groups (n = 5), or lipoprotein(a) concentrations were not reported quantitatively (n = 14). Ultimately, 17 studies satisfied all eligibility criteria and were included in the final synthesis.

### Characteristics of included studies

**Fig 2:**
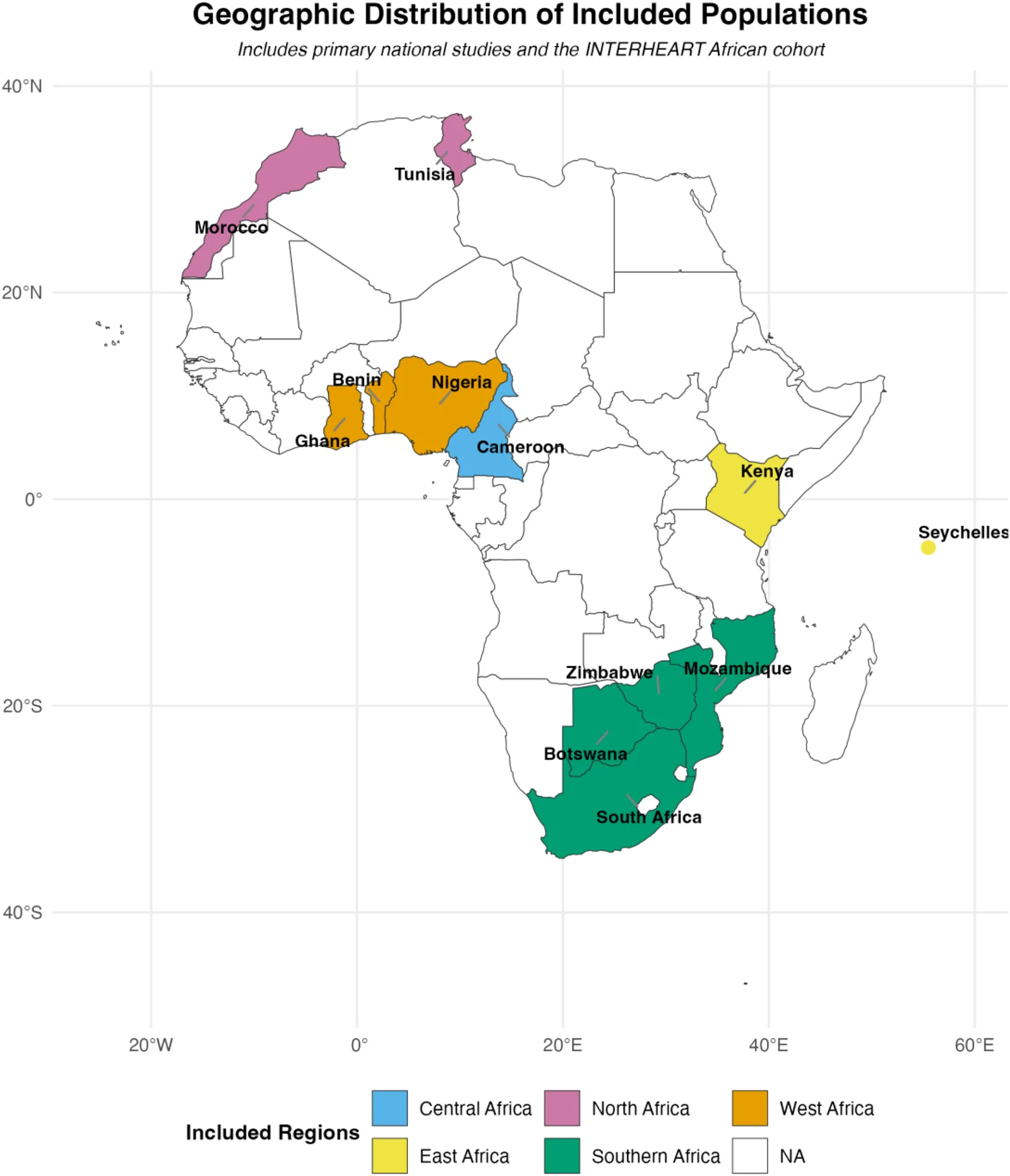
Geographic distribution of populations represented in included studies, (The map shows the African countries represented by primary national studies and the African cohort of the INTERHEART study. Colours group included populations by African region: Central, East, North, Southern, and West Africa. Unshaded countries were not represented by a primary national population in the included studies), eight studies were conducted in West Africa, including six from Nigeria [16–21] and three from Ghana [22–24]; one study originated from Central Africa [25] (Cameroon); five studies were conducted in Southern Africa, predominantly in South Africa [26–28] with one from Zimbabwe [29]; and two studies were conducted in North Africa, from Morocco [30] and Tunisia [31]. Only one study was conduct across all regions [32] in countries such as Benin, Botswana, Cameroon, Kenya, Mozambique, Nigeria, Seychelles, South Africa and Zimbabwe. Most included studies employed cross-sectional designs [17,18,21–23,25–27], often involving surveys of defined population groups, or case–control designs [16,19,20,29–32] in which apparently healthy individuals were recruited as controls for patients with conditions such as myocardial infarction, stroke, or diabetes. Sample sizes of the healthy control groups varied widely, ranging from approximately 50 participants in early pilot studies to more than 1,400 participants in more recent large-scale studies. Across studies, participants were predominantly adults aged between 18 and 70 years, with recruitment settings varying from blood donor clinics to hospital staff populations and community-based samples. The quantification of Lp(a) levels across the included studies relied predominantly on enzyme-linked immunosorbent assays (ELISA), which were utilized in 12 of the 17 studies, particularly those conducted between 1992 and 2020 [16–20,22,23,25,27,29,30]. A clear methodological evolution is evident in the most recent literature, with large-scale investigations such as Bruwer and colleagues [28] and also Eguvbe and colleagues [21]. (2024) employing automated particle-enhanced immunoturbidimetric assays. Other methods included immunoradiometric assays (IRMA) [26].

The 17 studies included in the qualitative synthesis were published between 1992 and 2024. Key characteristics of the included studies are summarized in Table 1. Geographically as shown in Fig 2.

**Table 1:** Summary of Included Studies and Lp(a) Levels in Apparently Healthy African Populations.

S/N	Study ID	Country (Region)	Study Design	Sample Size (N)	Study Population & Setting	Assay Method
	**West Africa**					
1	Cobbaert & Kesteloot (1992) [16]	Nigeria	Case-Control	60	Healthy volunteers (Benin City)	ELISA
2	Rotimi et al. (1997) [17]	Nigeria	Cross-Sectional	252	Community sample (Ibadan)	ELISA
3	Evans et al. (1997) [18]	Nigeria	Cross-Sectional	809	Civil servants (Benin City)	ELISA
4	Ebesunun et al. (2007) [19]	Nigeria	Case-Control	170	CVD patients & controls (Ibadan)	ELISA
5	Ogbera & Azenabor (2010) [20]	Nigeria	Case-Control	300	Type 2 Diabetes patients & controls	ELISA
6	Appiah et al. (2020) [22]	Ghana	Cross-Sectional	147	People Living with HIV (PLWH) and healthy controls	ELISA
7	Gyabaah et al. (2024) [23]	Ghana	Cross-Sectional	116	Stroke survivors (Kumasi)	ELISA
8	Eguvbe et al. (2024) [21]	Nigeria	Cross-Sectional	300	Hypertensive & Normotensive adults	Immunoturbidimetry
9	Li et al. (2020) [24]	Ghana	Cross-Sectional	1106	General Population	ELISA
	**Southern Africa**					
10	Carstens et al. (1998) [26]	South Africa	Cross-Sectional	111	Black male factory workers	IRMA
11	Dube et al. (2002) [29]	Zimbabwe	Case-Control	84	Healthy blood donors (Harare)	ELISA
12	Choma et al. (2003) [27]	South Africa	Cross-Sectional	90	Healthy villagers (Dikgale)	ELISA
13	Bruwer et al. (2024) [28]	South Africa	Cohort	1,462	Healthy adults (Prospective Urban and Rural Epidemiology [PURE] Study)	Immunoturbidimetry
	**Central Africa**					
14	Cobbaert et al. (1997) [25]	Cameroon	Cross-Sectional	354	Pygmies and Bantus	ELISA
	**North Africa**					
15	Fellat et al. (1997) [30]	Morocco	Case-Control	84	Hospitalized coronary patients/controls	ELISA
16	Smaoui et al. (2003) [31]	Tunisia	Case-Control	300	Type 2 Diabetes patients & controls	Nephelometry
	**Multi-Regional**					
17	Paré et al. (2019) [32]	Benin, Botswana, Cameroon, Kenya, Mozambique, Nigeria, Seychelles, South Africa and Zimbabwe	Case-Control	481	INTERHEART African cohort	Immunoassay

### Methodological quality and risk of bias

The risk of bias assessment (summarized in Table 2) of the 17 included studies had nine studies classified as low risk [19–23,25,28,32] and seven as moderate risk [16–18,26,27,29,30]. significant methodological heterogeneity, particularly regarding the validity of the outcome measurement. No studies were excluded based on high risk (scores <5). A temporal trend in methodological rigor was observed across the included studies. Investigations published in the last decade consistently achieved higher quality scores. In contrast, earlier studies published between 1992 and 2003 were more frequently classified as moderate risk.

**Table 2:** Risk of Bias Summary (Newcastle-Ottawa Scale).

Study ID	Study Design†	Selection (Max 4)	Comparability (Max 2)	Outcome / Exposure (Max 3)	Total Score	Risk of Bias
**West Africa**						
Cobbaert & Kesteloot (1992)	Cross-Sectional	★★★	☆☆	★★	5	Moderate
Rotimi et al. (1997)	Cross-Sectional	★★★	☆☆	★★★	6	Moderate
Evans et al. (1997)	Cross-Sectional	★★★	☆☆	★★★	6	Moderate
Ebesunun et al. (2007)	Case-Control	★★★★	☆☆	★★★	7	Low
Ogbera & Azenabor (2010)	Cross-Sectional	★★★	★★	★★★	8	Low
Appiah et al. (2020)	Cross-Sectional	★★★	★★	★★★	8	Low
Gyabaah et al. (2024)	Cross-Sectional	★★★	★★	★★★	8	Low
Eguvbe et al. (2024)	Cross-Sectional	★★★	★	★★★	7	Low
Li et al. (2020)	Cross-Sectional	★★★	★★	★★★	8	Low
**Southern Africa**						
Carstens et al. (1998)	Cross-Sectional	★★★	☆☆	★★	5	Moderate
Dube et al. (2002)	Cross-Sectional	★★★	☆☆	★★	5	Moderate
Choma et al. (2003)	Cross-Sectional	★★★	☆☆	★★	5	Moderate
Bruwer et al. (2024)	Cohort	★★★★	★★	★★★	9	Low
**North & Central Africa**						
Cobbaert et al. (1997)	Cross-Sectional	★★★	★	★★★	7	Low
Fellat et al. (1997)	Cross-Sectional	★★★	☆☆	★★★	6	Moderate
Smaoui et al. (2003)	Case-Control	★★★★	☆☆	★★★	7	Low
**Multi-Regional**						
Paré et al. (2019)	Case-Control	★★★★	★	★★★	8	Low

Key: ★ = Point awarded; ☆ = Point not awarded.

† Study Design: Corresponds to the specific NOS tool version applied during the risk of bias assessment (For Cross-Sectional studies, Selection is out of 5 stars. For Case-Control/Cohort, Selection is out of 4 stars.).

Thresholds: Total scores of 7–9 indicate Low Risk/Good Quality; scores of 5–6 indicate Satisfactory/Moderate Risk; scores <5 indicate High Risk.

### Prevalence and distribution of Lp(a) in Africa

#### Regional variations.

**Fig 3:**
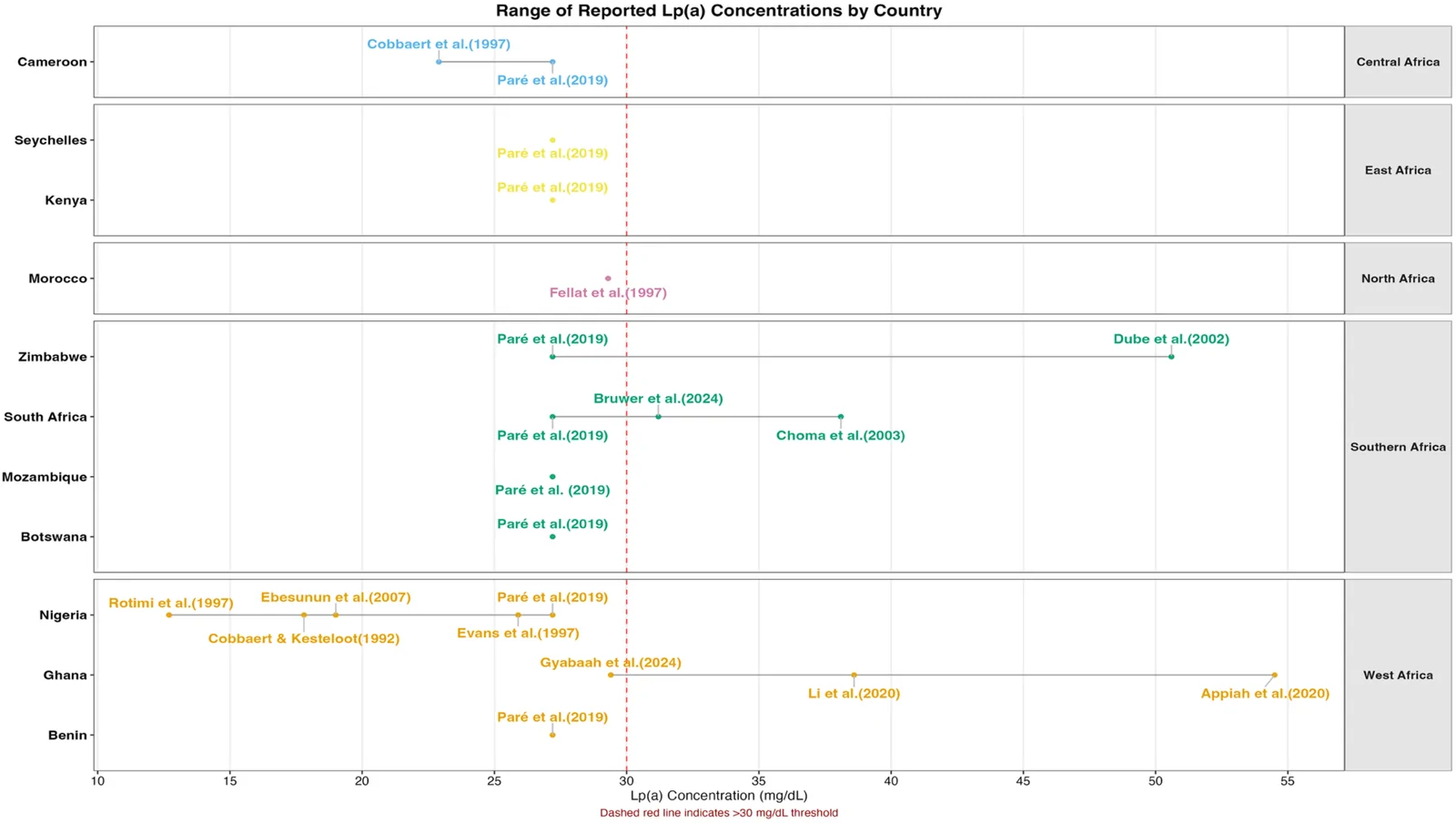
Ranges of reported lipoprotein(a) concentrations across included African populations. Study-specific lipoprotein(a) values and reported ranges are displayed by country and grouped by African region. Points denote reported concentrations; horizontal lines connect the lower and upper values where a range was available. The dashed red line marks 30 mg/dL. Lp(a), lipoprotein(a).

A consistent finding across the review was the high burden of elevated Lp(a) in West African populations compared to other regions (see Fig 3).

From West Africa, studies in Nigeria and Ghana consistently reported high mean and median values. Rotimi et al. (1997) found a mean of 12.7 mg/dL in a community sample in Ibadan [17] while a mean of 29.4 mg/dL [23] and 54.5 mg/dL [22] was identified in Ghana. Similarly, the INTERHEART study, which included a substantial African cohort, identified Africans as having the highest median Lp(a) concentration of 27.2 mg/dL [32]. The distribution in Southern Africa revealed a mean of 38.1 mg/dL in south Africa. However, the recent large-scale PHURE study in South Africa reported a median of 31.2 mg/dL [28]. In North African populations, a mean of 26.9 mg/dL was reported in healthy Tunisian controls was reported

### The threshold and elevation of Lp(a)

The estimation of elevated [Lp(a)] prevalence across African populations as shown in Table 3 indicates considerable variability depending on the cut-off applied and the study population. Using the conventional threshold of ≥30 mg/dL, studies in West and Southern Africa reported moderate to high prevalence levels. In Ghana approximately 30% was reported among stroke survivors [23] and 61% among PLHIV on Anti-Retroviral Treatment (ART) [22]. Among healthy participants of a study in Nigeria, 4% of the population had elevated Lp(a) [20]. A higher prevalence of 55% was reported in central Africa (Cameroon) [25].

**Table 3:** Lp(a) Concentrations and Prevalence of Elevated Levels.

	Region	Study (Year)	Population (Sub-group)	Reported Central Value (Original Unit)	Converted/Standardized Value (mg/dL)*	Overall Prevalence of Elevated Lp(a)
	West Africa					
1		Appiah et al. (2020)	PLWH	54.5 mg/dL	54.5 mg/dL	61% (>30 mg/dL)
2		Cobbaert & Kesteloot (1992)	Healthy Volunteers	178 mg/L (Mean)	17.8 mg/dL	NR
3		Ebesunun et al. (2007)	Controls	0.45 µmol/L	~19.0 mg/dL	NR
4		Eguvbe et al. (2024)	Normotensive	NR	NR	NR
5		Evans et al. (1997)	Civil Servants	25.9 mg/dL	25.9 mg/dL	NR
6		Gyabaah et al. (2024)	Stroke Survivors	29.4 mg/dL (Mean)	29.4 mg/dL	30.2% (>30 mg/dL)
7		Ogbera & Azenabor (2010)	Controls	NR	NR	4% (>30 mg/dL)
8		Rotimi (1997)	Community	12.7 mg/dL (Mean)	12.7 mg/dL	NR
9		Li et al. (2020)	General Population	38.6 mg/dL (Median)	38.6 mg/dL	NR
	Southern Africa					
10		Bruwer et al. (2024)	Healthy (PURE)	67.1 nM (Median)	31.2 mg/dL	NR
11		Carstens et al. (1998)	Factory Workers	NR	NR	70.6% (>300 U/L)
12		Choma et al. (2003)	Healthy (Rural)	38.1 mg/dL (Mean)	38.1 mg/dL	NR
13		Dube et al. (2002)	Healthy Donors (Blacks)	506 mg/L (Mean)	50.6 mg/dL	NR
	North Africa					
14		Fellat et al. (1997)	Controls	293 mg/L	29.3 mg/dL	NR
15		Smaoui et al. (2003)	Controls	269.83 mg/L (Mean)	26.9 mg/dL	26% (>30 mg/dL)
	Central Africa					
16		Cobbaert et al. (1997)	Bantu (Farmers) Pygmies (Hunter-gatherers)	229 mg/L	22.9 mg/dL	55% (>30 mg/dL)
	**Multi-Regional**					
17		Paré et al. (2019)	African Cohort	27.2 mg/dL (Median)	27.2 mg/dL	27% (> 50 mg/dL)

Note on Conversion:

mg/L to mg/dL: Divided by 10.

nmol/L to mg/dL: Divided by approximately 2.15 (common conversion factor for Lp(a), though exact conversion depends on isoform size).

µmol/L to mg/dL: Converted to nmol/L (x1000) then divided by 2.15.

### Determinants of Lp(a) variability

#### Demographic factors.

The influence of demographic variables, particularly gender, on Lp(a) concentrations showed consistent trends across several studies (Table 4). A female preponderance for higher Lp(a) levels was frequently observed. Evans et al. (1997) reported higher mean concentrations in female Nigerian civil servants (27.0 mg/dL) compared to their male counterparts (24.7 mg/dL). This observation was reinforced by more recent studies; Gyabaah et al. (2024) found that female sex was significantly associated with elevated Lp(a) in Ghanaian stroke survivors (OR: 3.09), and Eguvbe et al. (2024) reported that females had 3.6 times higher odds of elevated Lp(a) than males in a Nigerian cohort. In North Africa, Fellat et al. (1997) also reported higher mean levels among females compared to males. However, other studies did not demonstrate or report statistically significant sex differences. Age, where assessed, showed no consistent association with Lp(a) levels.

**Table 4:** Determinants of Lp(a) Variability (Demographic, Anthropometric, and Genetic).

Region	Study (Year)	Gender Association (Male vs Female)	Conversion to mg/dL	Age Association	BMI / Obesity Association	Isoform Association
**West Africa**	Cobbaert & Kesteloot (1992)	Male: 37.9 ± 14 mg/L; Female: 33.5 ± 10.2 mg/L	M: 3.79 ± 1.4 mg/dL; F: 3.35 ± 1.02 mg/dL	NR	NR	NR
	Rotimi et al. (1997)	NR		NR	NR	Inverse relationship: smaller apo(a) isoform size associated with higher Lp(a) levels
	Evans et al. (1997)	Females: 27.0 ± 17.7 mg/dL); Males: 24.7 ± 18.2 mg/dL		No significant association	No significant association	NR
	Ebesunun et al. (2007)	NR		NR	NR	NR
	Ogbera & Azenabor (2010)	NR		NR	NR	NR
	Appiah et al. (2020)	NR		NR	NR	NR
	Li et al. (2020)	Female: 38.7 mg/dL Male: 173 mg/dL		NR	NR	NR
	Gyabaaha et al. (2024)	Female sex significantly associated with elevated Lp(a) (OR: 3.09; 95% CI: 1.05–9.12)		NR	NR	NR
	Eguvbe et al. (2024)	Female sex associated with elevated Lp(a) (OR: 3.6; 95% CI: 1.4–9.8)		NR	Obesity associated with elevated Lp(a) (OR: 9.3; 95% CI: 2.5–34.0)	NR
**Southern Africa**	Carstens et al. (1998)	Not applicable (all-male study population)		NR	NR	Decreasing frequency of single-band apo(a) phenotypes with increasing Lp(a); double-band increased with higher levels
	Dube et al. (2002)	Male: 545 ± 687 mg/L; Female: 461 ± 451 mg/L	Male: 54.5 ± 68.7 mg/dL; Female: 46.1 ± 45.1 mg/dL	NR	NR	Smaller isoforms (e.g., S4) associated with higher Lp(a) concentrations
	Choma et al. (2003)	NR		NR	NR	No association between Lp(a) levels and apo(a) isoforms
	Bruwer et al. (2024)	NR		NR	NR	Not reported
**Central Africa**	Cobbaert et al. (1997)	NR		NR	NR	No clear isoform association reported; similar Lp(a) levels across Pygmy and Bantu groups
**North Africa**	Fellat et al. (1997)	Females (310 ± 101 mg/L)> Males (275 ± 120 mg/L)	Females (31.0 ± 10.1 mg/dL)> Males (27.5 ± 12.0 mg/dL)	NR	NR	NR
	Smaoui et al. (2003)	NR		NR	NR	NR
**Multi-Regional**	Paré et al. (2019)	NR		NR	NR	Africans had the smallest apo(a) isoform size (median 24 KIV repeats) and higher Lp(a) concentrations compared to other ethnic groups

Summary of associations reported in included studies. “NR” indicates the study did not report statistical data for that specific variable.

#### Isoform size.

The genetic architecture of Lp(a) in African populations was characterized by a distinct relationship with apolipoprotein(a) [apo(a)] isoform size. Rotimi et al. (1997) confirmed that smaller apo(a) sizes correlated with higher Lp(a) levels in West Africa. Similarly, Dube et al. (2002) in Zimbabwe found that the S4 phenotype (a smaller isoform) was associated with higher concentrations compared to larger isoforms (>S4). On a broader scale, the INTERHEART study [32] highlighted that Africans possessed the smallest median isoform size (24 KIV repeats) among all ethnic groups studied, providing a genetic basis for the generally higher mean Lp(a) levels observed across the continent. However, the relationship is complex; Choma et al. (2003) reported no significant association in a rural South African cohort, and Carstens et al. (1998) noted specific phenotype distribution patterns.

### Associations with cardiometabolic risk factors

#### Clinical factors.

Associations between clinical anthropometric measures and Lp(a) were generally weak or inconsistent across the reviewed literature. A notable exception was the study by Eguvbe et al. (2024), which reported a strong association between obesity and elevated Lp(a) (OR: 9.3) in a Nigerian population. In contrast, a study by Evans and colleagues found no significant association between Lp(a) levels and anthropometric measures [18].

#### Hypertension and diabetes.

In the context of hypertension (see Table 5), Eguvbe et al. (2024) observed a marked elevation in Lp(a) levels among hypertensive patients (32.8 ± 16.6 mg/dL) compared to normotensive controls (16.9 ± 13.9 mg/dL). Similarly, a consistent link with Type 2 Diabetes Mellitus (T2DM) was identified. Ogbera & Azenabor (2010) reported significantly higher mean Lp(a) concentrations in Nigerian T2DM patients compared to controls (56.1 vs. 28.6 mg/dL). This finding was corroborated by Smaoui et al. (2003) in Tunisia, who found that 46% of diabetic patients had elevated Lp(a) compared to only 26% of controls. Furthermore, Gyabaah et al. (2024) identified diabetes as a significant independent predictor of elevated Lp(a) among stroke survivors (OR: 3.52).

**Table 5:** Association of Lp(a) with Cardiovascular Conditions and Risk Factors in African Populations.

Study	Risk Factor Category	Specific Risk Factor	Key Findings & Direction of Association	Statistical Estimate / Effect Size
Eguvbe et al. (2024)	Hypertension	Systemic Hypertension	Lp(a) levels were significantly higher in hypertensive individuals compared to normotensive controls.	Hypertensive: 32.8 ± 16.6 mg/dL; Control: 16.9 ± 13.9 mg/dL
Ogbera & Azenabor (2010)	Diabetes Mellitus	Type 2 Diabetes Mellitus (T2DM)	T2DM patients had substantially higher mean Lp(a) levels than controls.	T2DM: 56.1 ± 11.2 mg/dL; Control: 28.6 ± 5.9 mg/dL
Smaoui et al. (2003)	Diabetes Mellitus	T2DM	Higher prevalence of elevated Lp(a) among diabetic patients compared to non-diabetics.	Elevated Lp(a) (>30 mg/dL): T2DM: 46% vs Control: 26%
Gyabaah et al. (2024)	Diabetes Mellitus	T2DM of Ischemic Stroke survivors	Diabetes independently predicted elevated Lp(a) among stroke survivors.	OR: 3.52 (95% CI: 1.32–9.40)
	Demographics	Resisdence of Ischemic Stroke survivors	Approximately one-third of stroke survivors had elevated Lp(a); urban residence was also a strong predictor.	Urban residence: OR: 4.64 (95% CI: 1.61–13.39)
Paré et al. (2019)	Cardiovascular Events	Myocardial Infarction	Higher Lp(a) concentrations were associated with increased risk of myocardial infarction in a multi-ethnic cohort.	OR: 1.09 (95% CI: 0.70–1.70)
Fellat et al. (1997)	Cardiovascular Events	Coronary Artery Disease	Lp(a) levels were positively correlated with coronary risk severity, independent of other risk factors.	Not reported
Bruwer et al. (2024)	Thrombosis	Clot Formation & Structure	Positive association between Lp(a) levels and clot density and rate of formation.	Significant correlation (p < 0.05)
Appiah et al. (2020)	Infectious Disease / CVD Risk	HIV Infection	Elevated Lp(a) prevalent among PLWH; ART exposure modified cardiovascular risk profile.	No ART: OR: 0.46 (0.25–0.84); On ART: OR: 2.67 (1.53–4.68)
Evans et al. (1997)	Lifestyle Factors	Smoking, Alcohol, Anthropometry	No significant association between Lp(a) and traditional lifestyle or anthropometric risk factors.	No significant correlation

*Acronyms: OR = Odds Ratio; HTN = Hypertension; T2DM = Type 2 Diabetes Mellitus;*

*PLWH = People Living with HIV; NR = Not Reported quantitatively in extracted summary.*

### Cardiovascular events and thrombotic mechanisms

The review highlighted the contribution of Lp(a) to acute cardiovascular events and thrombotic risk in African populations. In the INTERHEART study, Paré et al. (2019) observed a trend where higher Lp(a) concentrations were associated with an increased risk of myocardial infarction in the African cohort by 9%. Specific to cerebrovascular disease, Gyabaah et al. (2024) reported that approximately one in three Ghanaian ischemic stroke survivors harbored elevated Lp(a), with urban residence emerging as a potent predictor (OR: 4.64). Beyond atherosclerosis, Bruwer et al. (2024) provided mechanistic insight, demonstrating a positive association between Lp(a) concentration and both the rate of clot formation and clot density. Additionally, Appiah et al. (2020) noted a 2.67 higher risk of Lp(a) above ≥ 30 mg/dl among PLWH on ART in Ghana.

## Discussion

### Summary of main findings

This systematic review consolidates data from over three decades of research, confirming that elevated Lp(a) is a highly prevalent trait in individuals of sub-Saharan African descent. This synthesis reveals a distinct geographic pattern, with West African populations (Nigeria [20], Ghana [22,23]) generally exhibiting high mean concentrations (25−25 mg/dL) compared to other African cohorts. Using the commonly applied cut-off of 30 mg/dL, prevalence of increased Lp (a) concentrations ranged between 30% and 61%. Furthermore, the findings highlight a significant gender disparity, with females frequently presenting with higher Lp(a) concentrations than males, and a significant association with hypertension, T2DM and PLHIV on ART. Genetic evidence across studies highlights an inverse relationship between apo(a) isoform size and Lp(a) concentration, with smaller isoforms being associated with higher circulating Lp(a) levels.

### The African Lp(a) paradox

The historical notion that high Lp(a) in Africans is less atherogenic [25] is challenged by the robust associations with clinical outcomes identified in this review [22,23,32]. While some early studies focused on healthy cohorts [20,25], data from clinical populations demonstrate clear pathological risks [22,23,32]. Gyabaah et al. (2020) found that one in three ischemic stroke survivors in Ghana possessed elevated Lp(a), with urban residence increasing the odds approximately fourfold. Similarly, the INTERHEART African cohort showed a trend toward increased myocardial infarction risk with higher concentrations [32]. Furthermore, the recent cohort data from Bruwer et al. (2024) provides a plausible mechanism for this pathogenicity, demonstrating a direct correlation between Lp(a) levels and clot density [28]. Taken together, these findings suggest that rather than being inherently protective, elevated Lp(a) in African populations likely confers significant cardiovascular risk, which may become more pronounced in the presence of coexisting metabolic and environmental exposures.

### Lp(a) threshold in the African context

The application of conventional Lp(a) thresholds derived from non-African populations is increasingly questionable when evaluated against the distribution patterns observed across African cohorts in this review. Using the widely adopted cut-off of >30 mg/dL, studies across West [22,23], Central [25], and Southern Africa [26] classify a substantial proportion of ostensibly healthy individuals as having elevated Lp(a), with prevalence estimates frequently ranging between 30% and 61%. The population-based data across Africa indicate that nearly one out of four of participants exceed the threshold of 50 mg/dL [32]. This consistently right-shifted distribution suggests that Lp(a) levels in African populations are inherently higher, likely reflecting underlying genetic architecture [1,5,8,33]. It is important to distinguish between continental African populations and diaspora groups such as African Americans and Afro-Caribbeans. While these diaspora populations also exhibit elevated Lp(a) levels, their genetic profiles reflect significant admixture with European, Indigenous American, and other ancestries [33,34]. They are also exposed to markedly different environmental contexts including dietary patterns, physical activity levels, and urbanization trajectories than populations residing in continental Africa [35,36]. Similarly, studies show that African migrants in high-income countries often experience dietary acculturation, with younger and more recent migrants more readily adopting Western dietary patterns characterized by energy-dense, fat-rich foods [37], and face challenges in maintaining physical activity levels [38]. Consequently, risk thresholds and clinical interpretations derived from diaspora studies may not be directly transferable to locals settings such as in Ghana or Nigeria. Consequently, applying a uniform binary threshold risk may either overestimate the abnormality or may fail to appropriately stratify the cardiovascular risk. These findings reflect the need for context-specific reference ranges or risk-based thresholds that better reflect the baseline distribution and clinical relevance of Lp(a) in African populations rather than reliance on European population-based cut-offs.

### Methodological standardization

The risk of bias assessment of this review revealed a clear temporal shift in methodology that impacts the interpretation of results. Older studies (1992–2003) predominantly relied on ELISA methods, which are often sensitive to apo(a) isoform size. Given that African populations exhibit the smallest median isoform sizes globally (24 Kringle IV type 2 [KIV-2] repeats), as noted by Paré et al. (2019) [32], these older assays may have introduced systematic bias. While KIV-2 repeats explain the vast majority of the variance in Lp(a) levels in European populations (often >70%), this specific genetic trait explains a considerably smaller proportion of the variance in Africans. Because Lp(a) concentrations are less strictly dictated by KIV-2 genetics in African cohorts [1,5,6], relying on older assays that use antibodies directed at these repeats introduces systematic bias and likely underestimates true Lp(a) mass. The transition to isoform-insensitive immunoturbidimetric assays in recent high-quality studies [21,28] provides more reliable quantification. To ensure accurate risk stratification, laboratories across Africa should adopt assays calibrated against WHO/IFCC reference standards and report results in nmol/L, which eliminates the bias introduced by apo(a) isoform size variability and further enables valid cross-population and cross-study comparisons. This underscores that future research in the region must abandon unstandardized immunoassays in favour of methods traceable to WHO/IFCC standards to accurately map the continent’s risk profile [39].

### Clinical and public health implications

From a clinical and public health perspective, the findings suggest that while universal Lp(a) screening may not be immediately feasible in many African settings, a targeted, risk-based approach is both practical and potentially high-yield. However, for screening to be clinically valid, laboratories must emphasize the absolute necessity of utilizing isoform-independent assays and results in nmol/L. Furthermore, African clinicians must exercise caution regarding the clinical application of Western diagnostic thresholds. Given the right-shifted distribution and naturally higher baseline Lp(a) concentrations inherent to African phenotypes, rigidly applying Western-derived cut-offs may over-pathologize the general population and mischaracterize individual relative risk. Until robust, continent-specific reference ranges and risk percentiles are established, clinicians should avoid using these Western cut-offs as binary diagnostic tools. Instead, Lp(a) should be interpreted contextually as a continuous risk amplifier.

Additionally, evidence across the reviewed studies consistently demonstrates strong associations between elevated Lp(a) and key cardiometabolic conditions, particularly hypertension and type 2 diabetes mellitus. Additionally, clinical cohorts demonstrate significantly increased odds of elevated Lp(a), reinforcing its role in established cardiovascular disease. These patterns suggest that Lp(a) testing should be prioritized among patients with hypertension, diabetes, and premature or unexplained cardiovascular events. Importantly, in the absence of widespread access to Lp(a)-lowering therapies, identifying elevated levels should prompt intensified management of modifiable risk factors, particularly blood pressure, glycemic control, and lipid profiles, given the likely synergistic effect between Lp(a) and these conditions in driving cardiovascular risk in African populations.

### Limitations

This study synthesized literature spanning over three decades (1992–2024) of epidemiological data on Lp(a) across diverse African populations. This extensive timeframe allows for the unique observation of both the temporal evolution in assay methodologies and the shifting cardiometabolic risk profiles on the continent. By rigorously evaluating study quality using the Newcastle-Ottawa Scale and deliberately stratifying the data by region, this review successfully identifies crucial geographical differences. However, the review is subject to some limitations driven by the primary data. The primary limitation of this review is the high degree of heterogeneity across the included studies. Because researchers employed a wide variety of study designs and utilized diverse laboratory assays and assay units, a formal meta-analysis was neither feasible nor scientifically sound, limiting our ability to provide a single, unified “African prevalence” figure. Many of the older studies included in this review did not specify whether their assays were calibrated against the current WHO/IFCC international reference standards. Also, most identified studies reported results in mass units (mg/dL) rather than molar concentration (nmol/L). While we applied standard conversion factors to allow for comparison, we recognize that this is an imperfect solution. In African populations, where genetic diversity and apolipoprotein(a) isoform size variation are at their highest globally, these universal conversions may introduce imprecision. The lack of isoform-independent assays in many of the earlier studies remains a notable caveat in interpreting absolute levels. Another notable limitation is the geographic imbalance; the data are heavily skewed toward West Africa (Nigeria, Ghana) and South Africa, representing over two-thirds of the included cohorts. This leaves a conspicuous “data desert” in East Africa and parts of Central Africa, meaning our findings may not fully capture the linguistic, ethnic, and genetic diversity of the entire continent. Finally, the majority of the available data is cross-sectional. Without longitudinal cohort studies, this review can describe the presence of elevated Lp(a) in Africa, but it cannot yet definitively quantify its long-term predictive value for cardiovascular events within these specific populations. Nevertheless, the identification of consistent associations with stroke and myocardial infarction across several studies underscores the importance of Lp(a) in the African context.

## Conclusion

Elevated Lp(a) is a common, genetically determined trait in African populations, with a burden that is particularly pronounced in West Africa and among women. Far from being benign, evidence links this elevation to increased risks of stroke, myocardial infarction, and pro-thrombotic states, with additional risk modification observed among PLWH on ART. As the continent faces a rising tide of hypertension and diabetes, the synergistic risk posed by high Lp(a) requires aggressive prevention strategies and further prospective studies. Particular emphasis should be placed on Central and Eastern Africa, where evidence remains sparse, to ensure that future guidelines reflect the full spectrum of continental diversity. Moving forward, the adoption of standardized, isoform-independent assays, reporting in nmol/L, and the establishment of population-specific risk thresholds are essential to effectively integrate Lp(a) into cardiovascular risk stratification in Africa.

## Supporting information

S1 Table Database Search Strategy and Yields. (DOCX)

S2 Table Data Extraction Table. (DOCX)

S3 Table Newcastle-Ottawa Scale adapted for cross-sectional studies. (DOCX)
