# Supplementary material for: The burden of Lipoprotein(a) [Lp(a)] in Africa: A systematic review
Source: PLoS One. 2026 Aug 26;21(8):e0356490. doi: 10.1371/journal.pone.0356490 (PMC13513974; doi:10.1371/journal.pone.0356490)
Supplement: Supplementary file 1 [file pone.0356490.s001.docx]

Supplementary Table 1:Database Search Strategy and Yields

| **Database** | **Date of Search** | **Search Strategy (Terms, Keys, and Boolean Operators)** | **Filters / Limits Applied** | **Yield (Hits)*** |
| --- | --- | --- | --- | --- |
| **PubMed (MEDLINE)** | 1-02-2025 | ("Lipoprotein(a)"[MeSH Terms] OR "Lp(a)"[Title/Abstract] OR "Lipoprotein a"[Title/Abstract]) **AND** ("Africa"[MeSH Terms] OR "Africa"[Title/Abstract] OR "Sub-Saharan Africa"[Title/Abstract] OR "Algeria"[Title/Abstract] OR "Angola"[Title/Abstract] OR "Benin"[Title/Abstract] OR "Botswana"[Title/Abstract] OR "Burkina Faso"[Title/Abstract] OR "Burundi"[Title/Abstract] OR "Cabo Verde"[Title/Abstract] OR "Cape Verde"[Title/Abstract] OR "Cameroon"[Title/Abstract] OR "Central African Republic"[Title/Abstract] OR "Chad"[Title/Abstract] OR "Comoros"[Title/Abstract] OR "Congo"[Title/Abstract] OR "Democratic Republic of the Congo"[Title/Abstract] OR "Djibouti"[Title/Abstract] OR "Egypt"[Title/Abstract] OR "Equatorial Guinea"[Title/Abstract] OR "Eritrea"[Title/Abstract] OR "Eswatini"[Title/Abstract] OR "Swaziland"[Title/Abstract] OR "Ethiopia"[Title/Abstract] OR "Gabon"[Title/Abstract] OR "Gambia"[Title/Abstract] OR "Ghana"[Title/Abstract] OR "Guinea"[Title/Abstract] OR "Guinea-Bissau"[Title/Abstract] OR "Ivory Coast"[Title/Abstract] OR "Cote d'Ivoire"[Title/Abstract] OR "Kenya"[Title/Abstract] OR "Lesotho"[Title/Abstract] OR "Liberia"[Title/Abstract] OR "Libya"[Title/Abstract] OR "Madagascar"[Title/Abstract] OR "Malawi"[Title/Abstract] OR "Mali"[Title/Abstract] OR "Mauritania"[Title/Abstract] OR "Mauritius"[Title/Abstract] OR "Morocco"[Title/Abstract] OR "Mozambique"[Title/Abstract] OR "Namibia"[Title/Abstract] OR "Niger"[Title/Abstract] OR "Nigeria"[Title/Abstract] OR "Rwanda"[Title/Abstract] OR "Sao Tome and Principe"[Title/Abstract] OR "Senegal"[Title/Abstract] OR "Seychelles"[Title/Abstract] OR "Sierra Leone"[Title/Abstract] OR "Somalia"[Title/Abstract] OR "South Africa"[Title/Abstract] OR "South Sudan"[Title/Abstract] OR "Sudan"[Title/Abstract] OR "Tanzania"[Title/Abstract] OR "Togo"[Title/Abstract] OR "Tunisia"[Title/Abstract] OR "Uganda"[Title/Abstract] OR "Zambia"[Title/Abstract] OR "Zimbabwe"[Title/Abstract]) | Human, English Language | 255 |
| **Embase** | 1-02-2025 | ('lipoprotein a'/exp OR 'lipoprotein(a)':ti,ab OR 'lp(a)':ti,ab) **AND** ('africa'/exp OR 'africa':ti,ab OR 'sub saharan africa':ti,ab OR 'algeria':ti,ab OR 'angola':ti,ab OR 'benin':ti,ab OR 'botswana':ti,ab OR 'burkina faso':ti,ab OR 'burundi':ti,ab OR 'cabo verde':ti,ab OR 'cape verde':ti,ab OR 'cameroon':ti,ab OR 'central african republic':ti,ab OR 'chad':ti,ab OR 'comoros':ti,ab OR 'congo':ti,ab OR 'democratic republic of the congo':ti,ab OR 'djibouti':ti,ab OR 'egypt':ti,ab OR 'equatorial guinea':ti,ab OR 'eritrea':ti,ab OR 'eswatini':ti,ab OR 'swaziland':ti,ab OR 'ethiopia':ti,ab OR 'gabon':ti,ab OR 'gambia':ti,ab OR 'ghana':ti,ab OR 'guinea':ti,ab OR 'guinea-bissau':ti,ab OR 'ivory coast':ti,ab OR 'cote d''ivoire':ti,ab OR 'kenya':ti,ab OR 'lesotho':ti,ab OR 'liberia':ti,ab OR 'libya':ti,ab OR 'madagascar':ti,ab OR 'malawi':ti,ab OR 'mali':ti,ab OR 'mauritania':ti,ab OR 'mauritius':ti,ab OR 'morocco':ti,ab OR 'mozambique':ti,ab OR 'namibia':ti,ab OR 'niger':ti,ab OR 'nigeria':ti,ab OR 'rwanda':ti,ab OR 'sao tome and principe':ti,ab OR 'senegal':ti,ab OR 'seychelles':ti,ab OR 'sierra leone':ti,ab OR 'somalia':ti,ab OR 'south africa':ti,ab OR 'south sudan':ti,ab OR 'sudan':ti,ab OR 'tanzania':ti,ab OR 'togo':ti,ab OR 'tunisia':ti,ab OR 'uganda':ti,ab OR 'zambia':ti,ab OR 'zimbabwe':ti,ab) | Human, English | 200 |
| **Scopus** | 1-02-2025 | TITLE-ABS-KEY ("Lipoprotein(a)" OR "Lp(a)") **AND** TITLE-ABS-KEY ("Africa" OR "Sub-Saharan Africa" OR "Algeria" OR "Angola" OR "Benin" OR "Botswana" OR "Burkina Faso" OR "Burundi" OR "Cabo Verde" OR "Cape Verde" OR "Cameroon" OR "Central African Republic" OR "Chad" OR "Comoros" OR "Congo" OR "Democratic Republic of the Congo" OR "Djibouti" OR "Egypt" OR "Equatorial Guinea" OR "Eritrea" OR "Eswatini" OR "Swaziland" OR "Ethiopia" OR "Gabon" OR "Gambia" OR "Ghana" OR "Guinea" OR "Guinea-Bissau" OR "Ivory Coast" OR "Cote d'Ivoire" OR "Kenya" OR "Lesotho" OR "Liberia" OR "Libya" OR "Madagascar" OR "Malawi" OR "Mali" OR "Mauritania" OR "Mauritius" OR "Morocco" OR "Mozambique" OR "Namibia" OR "Niger" OR "Nigeria" OR "Rwanda" OR "Sao Tome and Principe" OR "Senegal" OR "Seychelles" OR "Sierra Leone" OR "Somalia" OR "South Africa" OR "South Sudan" OR "Sudan" OR "Tanzania" OR "Togo" OR "Tunisia" OR "Uganda" OR "Zambia" OR "Zimbabwe") | Article, English | 245 |
| **Web of Science** | 1-02-2025 | TS=("Lipoprotein(a)" OR "Lp(a)") **AND** TS=("Africa" OR "Sub-Saharan Africa" OR "Algeria" OR "Angola" OR "Benin" OR "Botswana" OR "Burkina Faso" OR "Burundi" OR "Cabo Verde" OR "Cape Verde" OR "Cameroon" OR "Central African Republic" OR "Chad" OR "Comoros" OR "Congo" OR "Democratic Republic of the Congo" OR "Djibouti" OR "Egypt" OR "Equatorial Guinea" OR "Eritrea" OR "Eswatini" OR "Swaziland" OR "Ethiopia" OR "Gabon" OR "Gambia" OR "Ghana" OR "Guinea" OR "Guinea-Bissau" OR "Ivory Coast" OR "Cote d'Ivoire" OR "Kenya" OR "Lesotho" OR "Liberia" OR "Libya" OR "Madagascar" OR "Malawi" OR "Mali" OR "Mauritania" OR "Mauritius" OR "Morocco" OR "Mozambique" OR "Namibia" OR "Niger" OR "Nigeria" OR "Rwanda" OR "Sao Tome and Principe" OR "Senegal" OR "Seychelles" OR "Sierra Leone" OR "Somalia" OR "South Africa" OR "South Sudan" OR "Sudan" OR "Tanzania" OR "Togo" OR "Tunisia" OR "Uganda" OR "Zambia" OR "Zimbabwe") | English | 150 |
| **African Journals OnLine (AJOL)** | 1-02-2025 | "Lipoprotein(a)" OR "Lp(a)" | None | 4 |
| **Total Yield** |  |  |  | **854** |
