# Supplementary material for: The burden of Lipoprotein(a) [Lp(a)] in Africa: A systematic review
Source: PLoS One. 2026 Aug 26;21(8):e0356490. doi: 10.1371/journal.pone.0356490 (PMC13513974; doi:10.1371/journal.pone.0356490)
Supplement: Supplementary file 2 [file pone.0356490.s002.docx]

Supplementary Table 2: Data Extraction Table

| Study (Author, Year) | Country (Region) | Study Design | Study Population & Setting | Sample Size (N) | Assay Method & Unit | Lp(a) Central Value (Mean/Median) | Prevalence of Elevated Lp(a) | **Gender Association (Male vs Female)** | **Age Association** | **BMI / Obesity Association** | **Genetic/Isoform Findings** | Risk Factor and Statistical estimate/association | Main Conclusion / Key Finding |
| --- | --- | --- | --- | --- | --- | --- | --- | --- | --- | --- | --- | --- | --- |
|  |  |  |  |  |  |  |  |  |  |  |  |  |  |
|  |  |  |  |  |  |  |  |  |  |  |  |  |  |
|  |  |  |  |  |  |  |  |  |  |  |  |  |  |
|  |  |  |  |  |  |  |  |  |  |  |  |  |  |
|  |  |  |  |  |  |  |  |  |  |  |  |  |  |
|  |  |  |  |  |  |  |  |  |  |  |  |  |  |
|  |  |  |  |  |  |  |  |  |  |  |  |  |  |
|  |  |  |  |  |  |  |  |  |  |  |  |  |  |
|  |  |  |  |  |  |  |  |  |  |  |  |  |  |
|  |  |  |  |  |  |  |  |  |  |  |  |  |  |
|  |  |  |  |  |  |  |  |  |  |  |  |  |  |
|  |  |  |  |  |  |  |  |  |  |  |  |  |  |
|  |  |  |  |  |  |  |  |  |  |  |  |  |  |
|  |  |  |  |  |  |  |  |  |  |  |  |  |  |
|  |  |  |  |  |  |  |  |  |  |  |  |  |  |
|  |  |  |  |  |  |  |  |  |  |  |  |  |  |
