# Supplementary material for: The burden of Lipoprotein(a) [Lp(a)] in Africa: A systematic review
Source: PLoS One. 2026 Aug 26;21(8):e0356490. doi: 10.1371/journal.pone.0356490 (PMC13513974; doi:10.1371/journal.pone.0356490)
Supplement: Supplementary file 3 [file pone.0356490.s003.docx]

Supplementary Table 3: Newcastle-Ottawa Scale adapted for cross-sectional studies

| Component | Scale |
| --- | --- |
| Selection: | 1. Representativeness of the sample:  a. Truly representative of the average in the target population. * (all subjects or  random sampling)  b. Somewhat representative of the average in the target group. * (non-random  sampling)  c. Selected group of users/convenience sample.  d. No description of the derivation of the included subjects.  2. Sample size:  a. Justified and satisfactory (including sample size calculation). *  b. Not justified.  c. No information provided  3. Non-respondents:  a. Proportion of target sample recruited attains pre-specified target or basic  summary of non-respondent characteristics in sampling frame recorded. *  b. Unsatisfactory recruitment rate, no summary data on non-respondents.  c. No information provided  4. Ascertainment of the exposure (risk factor):  a. Vaccine records/vaccine registry/clinic registers/hospital records only. **  b. Parental or personal recall and vaccine/hospital records. *  c. Parental/personal recall only. |
| Comparability: (Maximum 2 stars) | 1. Comparability of subjects in different outcome groups on the basis of design or  analysis. Confounding factors controlled.  a. Data/ results adjusted for relevant predictors/risk factors/confounders e.g.  age, sex, time since vaccination, etc. **  b. Data/results not adjusted for all relevant confounders/risk factors/information  not provided. |
| Outcome: | 1. Assessment of outcome:  a. Independent blind assessment using objective validated laboratory methods.  **  b. Unblinded assessment using objective validated laboratory methods. **  c. Used non-standard or non-validated laboratory methods with gold standard. *  d. No description/non-standard laboratory methods used.  2. Statistical test:  a. Statistical test used to analyse the data clearly described, appropriate and  measures of association presented including confidence intervals and  probability level (p value). *  b. Statistical test not appropriate, not described or incomplete. |
