# Supplementary material for: The burden of Lipoprotein(a) [Lp(a)] in Africa: A systematic review
Source: PLoS One. 2026 Aug 26;21(8):e0356490. doi: 10.1371/journal.pone.0356490 (PMC13513974; doi:10.1371/journal.pone.0356490)
Supplement: Supplementary file 4 [file pone.0356490.s005.docx]

| **Reviewer#** | **Exact Comment** | **Response** |
| --- | --- | --- |
| **Editor** | "This manuscript addresses a genuinely important and underexplored topic—the burden of Lp(a) in continental African populations. The authors have compiled a comprehensive dataset spanning three decades and multiple regions. The work is timely and potentially impactful. However, the authors must address some minor concerns raised by one of our reviewers before the manuscript can realize its potential impact." | We thank the Editor for this positive assessment of our work and for the opportunity to revise. We have carefully considered all reviewer comments and have made targeted revisions to strengthen the manuscript. A detailed point-by-point response is provided below. |
| **Editor** | 1. Please ensure that your manuscript meets PLOS ONE's style requirements, including those for file naming. The PLOS ONE style templates can be found at  https://journals.plos.org/plosone/s/file?id=wjVg/PLOSOne_formatting_sample_main_body.pdf and https://journals.plos.org/plosone/s/file?id=ba62/PLOSOne_formatting_sample_title_authors_affiliations.pdf | Manuscript revised to meet the style requirement of PLOS ONE. |
| **Editor** | 2. Please provide a complete Data Availability Statement in the submission form, ensuring you include all necessary access information or a reason for why you are unable to make your data freely accessible. If your research concerns only data provided within your submission, please write "All data are in the manuscript and/or supporting information files" as your Data Availability Statement. | Data were synthesis using available lieterature online. Search strategy has been provided to aid reproducibility |
| **Editor** | 3. We note that Figure 2 in your submission contain [map/satellite] images which may be copyrighted. All PLOS content is published under the Creative Commons Attribution License (CC BY 4.0), which means that the manuscript, images, and Supporting Information files will be freely available online, and any third party is permitted to access, download, copy, distribute, and use these materials in any way, even commercially, with proper attribution. For these reasons, we cannot publish previously copyrighted maps or satellite images created using proprietary data, such as Google software (Google Maps, Street View, and Earth). For more information, see our copyright guidelines: http://journals.plos.org/plosone/s/licenses-and-copyright.  We require you to either (1) present written permission from the copyright holder to publish these figures specifically under the CC BY 4.0 license, or (2) remove the figures from your submission:  a. You may seek permission from the original copyright holder of Figure 2 to publish the content specifically under the CC BY 4.0 license.  We recommend that you contact the original copyright holder with the Content Permission Form (http://journals.plos.org/plosone/s/file?id=7c09/content-permission-form.pdf) and the following text:  “I request permission for the open-access journal PLOS ONE to publish XXX under the Creative Commons Attribution License (CCAL) CC BY 4.0 (http://creativecommons.org/licenses/by/4.0/). Please be aware that this license allows unrestricted use and distribution, even commercially, by third parties. Please reply and provide explicit written permission to publish XXX under a CC BY license and complete the attached form.”  Please upload the completed Content Permission Form or other proof of granted permissions as an "Other" file with your submission.  In the figure caption of the copyrighted figure, please include the following text: “Reprinted from [ref] under a CC BY license, with permission from [name of publisher], original copyright [original copyright year].”  b. If you are unable to obtain permission from the original copyright holder to publish these figures under the CC BY 4.0 license or if the copyright holder’s requirements are incompatible with the CC BY 4.0 license, please either i) remove the figure or ii) supply a replacement figure that complies with the CC BY 4.0 license. Please check copyright information on all replacement figures and update the figure caption with source information. If applicable, please specify in the figure caption text when a figure is similar but not identical to the original image and is therefore for illustrative purposes only.  The following resources for replacing copyrighted map figures may be helpful:  USGS National Map Viewer (public domain): http://viewer.nationalmap.gov/viewer/  The Gateway to Astronaut Photography of Earth (public domain): http://eol.jsc.nasa.gov/sseop/clickmap/  Maps at the CIA (public domain): https://www.cia.gov/library/publications/the-world-factbook/index.html and https://www.cia.gov/library/publications/cia-maps-publications/index.html  NASA Earth Observatory (public domain): http://earthobservatory.nasa.gov/  Landsat: http://landsat.visibleearth.nasa.gov/  USGS EROS (Earth Resources Observatory and Science (EROS) Center) (public domain): http://eros.usgs.gov/#  Natural Earth (public domain): http://www.naturalearthdata.com/ | Fig 2 was produced by authors using R. |
| **Editor** | 4. Please include captions for your Supporting Information files at the end of your manuscript, and update any in-text citations to match accordingly. Please see our Supporting Information guidelines for more information: http://journals.plos.org/plosone/s/supporting-information. | Supporting Information file has been provided at the end of your manuscript (befere references) |
| **Editor** | 5. If the reviewer comments include a recommendation to cite specific previously published works, please review and evaluate these publications to determine whether they are relevant and should be cited. There is no requirement to cite these works unless the editor has indicated otherwise. | All recommendations reviewed and considered |
| **Editor** | 6. Please review your reference list to ensure that it is complete and correct. If you have cited papers that have been retracted, please include the rationale for doing so in the manuscript text, or remove these references and replace them with relevant current references. Any changes to the reference list should be mentioned in the rebuttal letter that accompanies your revised manuscript. If you need to cite a retracted article, indicate the article’s retracted status in the References list and also include a citation and full reference for the retraction notice. | We thank the reviewer for this . All have been checked and updated accordingly |
| **Reviewer #1** | "This is a well-conducted systematic review that fills a major gap in our understanding of Lp(a) prevalence and heart disease risk in Africa. The authors followed strict research guidelines, searched widely across multiple databases, and clearly demonstrate that high levels of Lipoprotein(a) are surprisingly common across the continent—affecting up to 60% of people in some groups." | We thank the reviewer for this positive and encouraging feedback. We are pleased that the scope and rigor of our review have been well received. |
| **Reviewer #1** | "Authors showed that this is not a harmless trait; it is strongly linked to strokes, heart attacks, and other serious conditions, especially when combined with the rising rates of high blood pressure and diabetes." | We thank the reviewer for this comment. We are gratified that the clinical significance of elevated Lp(a) in the African context is clearly conveyed. |
| **Reviewer #1** | "There was clear geographical representation from Western and Southern Africa, however Central and Eastern Africa shoed very little studies related to Lp(a) which can be a recommended research direction for these geographical areas." | We thank the reviewer for highlighting this important evidence gap. We have strengthened the conclusion to capture this  Pg 27, line 533-535 |
| **Reviewer #1** | "One area that could strengthen the discussion is a clearer comparison with African Americans and Afro-Caribbeans. While these diaspora groups also have high Lp(a) levels, their genetic makeup is mixed with other populations, meaning risk cut-offs developed in the US or Caribbean may not directly apply to someone living in rural Ghana or Nigeria. Additionally, environmental factors like diet, physical activity, and urbanisation play out very differently in Africa compared to the diaspora. Highlighting this would help readers understand why Africa needs its own, separate reference ranges rather than borrowing numbers from overseas studies that might not be a good fit." | We thank the reviewer for this constructive suggestion. We have expanded the **Discussion** to include a clearer distinction between continental African populations and diaspora groups.  page 17, lines 437–448 |
| **Reviewer #1** | "The biggest technical challenge raised by this review is the way Lp(a) is measured in laboratories. Older tests do not work well for African populations because they are easily affected by the specific size of the Lp(a) particles, which are uniquely small in many African groups. This can give falsely low readings and hide the true risk. To address this, labs across Africa urgently need to switch to modern, standardised tests that are not influenced by particle size. They should also report results in nmol/L rather than mg/dL, as this removes the size bias altogether and allows for accurate comparisons across the continent and with the rest of the world." | We thank the reviewer for raising this critical technical point. We have strengthened the discussion sections to underscore the urgency of transitioning to modern, standardized assays and reporting in molar units.  page 25-26, lines 467–470; 478-479. |
| **Reviewer #2** | "The manuscript titled 'The Burden of Lipoprotein (a)[ Lp(a)] in Africa: A Systematic Review, provides information about the effects of elevated Lipoprotein (a) levels on the health status of Africans. The data from this systematic review is technically and scientifically sound and can be replicated. Moreover, the conclusions of this review was comprehensively and appropriately made based on the data presented in the results." | We thank the reviewer for this positive assessment of the technical and scientific rigor of our review. We are pleased that the conclusions are felt to be appropriately supported by the data. |
| **Reviewer #2** | "However, it will be much appreciated if the data synthesis and analysis section could be elaborated in much detail." | We thank the reviewer for this helpful suggestion. We have substantially expanded the **"Data Synthesis and Analysis"** section in the **Methodology**  page 7-8, lines 208–223 |
| **Reviewer #2** | "Data obtained through this study were made available by the study authors in the submitted manuscript. The manuscript was swell structured and written in clear, unambiguous language, which is easy to read and comprehend." | We thank the reviewer for these comments. We confirm that all data underlying this systematic review are contained within the manuscript and/or Supporting Information files. We are pleased that the manuscript structure and clarity meet the reviewer's expectations. |
